# On the correlation between training modalities and recovery stages in poststroke robotic rehabilitation of the upper limb: a systematic review

**DOI:** 10.1186/s12984-026-02005-7

**Published:** 2026-04-30

**Authors:** Francesca Alvaro, Michele Perrelli, Rocco Adduci, Francesco Tedesco, Domenico Mundo

**Affiliations:** University of Calabria, Arcavacata, Italy

**Keywords:** Robotic rehabilitation, Upper limb, Stroke, Phase of recovery, Training modality

## Abstract

**Background:**

Robot-assisted therapy for poststroke rehabilitation of the upper limb is rapidly spreading. The need comes from the reduced number of therapists and the aim of defining a more involving therapy for the patients. Previous studies report the effectiveness of robotic therapy to provide intensive, repetitive and task-specific rehabilitation, as well as the ability to provide different modalities of training. However, it is not always clear how these modes are implemented and how they are defined, since different labels are sometimes used. As a consequence, it is difficult to define a universal protocol to follow. This leads to non-comparable outcomes, making even harder for the therapists to understand which is the most efficient way of administering therapy. The proposed work aims at putting together available information reported in literature, linking two main variables influencing rehabilitation, i.e., poststroke stage and training modality, with the purpose of updating the state of the art, categorising and analysing the modalities involved, extracting the most effective relationships and approaches in terms of results reported in the scientific community.

**Methods:**

Scopus was chosen as reference database for the systematic review. The studies refer to the last decade, that is from the year of the latest review published in relation to the topic of interest. Studies clearly referencing training modalities and poststroke stages were included.

**Results:**

The assistive modality is the one that catches more attention in the scientific community, highlighting the tendency to prefer approaches in which the patients are more actively involved. In terms of relevance inferable from clinical scales reported in the included studies, the assistive modality appears to be the most effective in the chronic phase, active-assistive approaches during the subacute one, whereas no significant conclusions can be drawn for the acute stage. For what concerns robotic devices, some considerations can be drawn as well: exoskeletons are applied during the chronic phase predominantly, whereas end-effectors during the subacute one. No significant distinctions are detected in the acute stage. Improvements in ADLs are mostly achieved in experiments involving exoskeletons, but studies show that subjects may also benefit from end-effectors, when applied in earlier recovery stages.

**Conclusions:**

It is evident that some connections are present between training modalities and recovery stages, influencing the outcomes of experimental trials. Evaluation metrics exploited in tests report enhanced outcomes when the association between training modality and poststroke stage is optimized. Nevertheless, future developments will possibly extend this study to other factors that may have influenced outcomes, such as intensity of the exercises, frequency and duration of the therapy, and impairment severity. Moreover, a deeper analysis, incorporating investigations on daily clinical practice, would help to identify of the most effective approaches.

**Supplementary Information:**

The online version contains supplementary material available at 10.1186/s12984-026-02005-7.

## Background

The frequent occurrence of neuromotor diseases, especially of stroke, requires improvements and changes in the rehabilitation process. Stroke survivors are usually left with hemiparesis, leading to weakness, loss of coordination and abnormal compensatory behaviours [1], which hinder the return to a normal life of the affected subjects. A timely intervention is crucial for an enhanced therapy, since the chance of recovery declines overtime [2]. For this reason, a proper poststroke treatment is relevant for a greater healing. Focusing on impairments related to the upper limb, however, conventional therapy presents some issues due to the reduced number of therapists and their high physical involvement, which takes away a lot of time for the planning and analysis of the treatment itself [3]. Consequently, the focus has been recently moved to robotic rehabilitation. This approach guarantees repetitive, intensive and task-specific training [2, 4–6], actively involving the patient in the process. Robotic devices already proved effective in the recovery of motor functions and neuroplasticity [7, 8], although not always in the recovery of Activities of Daily Living (ADLs). Even if this aspect constitutes one of the main contributions for the regaining of the independence of the patient, it still represents the most uncertain side of the outcomes, since the mechanisms influencing the improvements of these activities are not fully clear yet. In some cases, the outcomes for a similar approach do not match and this could be related to several reasons, such as the different structure of the robots employed, the frequency and the modality of training and the stage of recovery. This means that the administration of therapy does not follow a specific protocol. For these reasons, potential recoveries of ADLs are sometimes not associated with any particular approach, the path to follow still remains unknown and it is not clear how motor plasticity and recovery can be further improved [9]. Some studies investigated the relationship between latency, intended in this context as time between stroke onset and treatment [10], and modality of training but, considering the wide diffusion of these robotic devices in the rehabilitative field and their variety of applications, these correlations may have changed since the last study [1]. Accordingly, the present work arises from the need of extending and updating the state of the art by answering to the question: *“in robotic rehabilitation for the upper limb*,* how does the choice of the training modalities influence the outcomes of rehabilitative trials based on the phase in which they are applied?”*. Starting from what already emerges concerning the modality-phase combination, this work proposes its own categorisation and description of the modalities exploited for the administration of therapy, taking into account the poststroke stage during which they are employed, as well as the particular cases of applications. With the aim of extracting some points in common between the two variables, the work aims to provide insights on scientific findings, paving the way for clinicians towards the decision-making process for the administration of the robotic rehabilitative therapy. A further insight is given also to the structures employed, either exoskeletons or end-effectors, in order to identify the existing relationship in terms of usage during the poststroke stages. Before going into the details of the proposed analysis, poststroke stages and training modalities are defined in the following subsections.

### Stages of stroke

The Stroke Recovery and Rehabilitation Roundtable [11] classifies the poststroke period as: hyper-acute ($\:<24$h), acute ($\:>24$h and $\:<7$ days), early subacute ($\:>7$ days and $\:<3$ months), late subacute ($\:>3$ months and $\:<6$ months) and chronic ($\:>6$ months). For the purposes of this study, the partition does not go into such a level of detail, and the classification considers three main stages: acute ($\:<7$ days), subacute ($\:>7\:$days and $\:<6$ months) and chronic ($\:>6$ months). During the first part of the poststroke period, the patient has higher levels of brain plasticity that tend to decrease over time, leading to the chronic phase, which is the one with less spontaneous recovery [12]. In view of this, the most intuitive approach could be to start the rehabilitative exercises as soon as possible, exploiting the ease of relearning. Yet, this is not as simple as it might seem since, during the acute phase, the subjects are extremely impaired and are not able to accomplish specific movements. The opposite situation occurs during the chronic phase, when the patient has possibly regained a good amount of motor capabilities, but the reduced brain plasticity makes it difficult to learn new tasks. Nevertheless, this does not hold in all situations and in some cases, the recovery is still possible. This could be related to the way how the treatment is administered, hence to the modality applied. To identify the most suitable approach based on the phase of recovery, an analysis on the occurrence and effects of training modalities is performed, to find the approach with more promising results. The main modalities taken into account for the proposed study are passive, assistive, active-assistive, active and resistive, involving both unilateral and bilateral rehabilitation.

### Modalities of training

The training modalities are classified based on the type of human-robot interaction [13] and, for each of them, both unilateral and bilateral rehabilitative exercises can be accomplished. *Unilateral therapy* refers to training of the paretic limb only, whereas the term *bilateral* implies the use of both arms for the rehabilitative tasks [14], in which the impaired limb follows the behaviour of the unimpaired one [15]. The existing literature does not cover recent developments on bilateral applications in robotic rehabilitation and tends to classify those applications in categories with different names with respect to the unilateral ones, making the nomenclature diverse, difficult to track and sometimes misleading. Novel methodologies are enriching the subfield of bilateral rehabilitative robots, generating applications in which the tasks, the robotic device or the control strategy may change, but the way how patient and robot interact always falls back to one of the classes reported in Table 1 and summarized as follows. In the passive modality, only the robot controls the movements and the impaired arm follows without intervention. The assistive mode provides the patient with a continuous help during exercises, while the active intentions of the subject are detected. Assistance can be provided by forces applied in the direction of a target to facilitate the execution of the tasks or by counterbalancing the weight of the paretic arm, thus supporting the patient in the accomplishment of the exercises. In the bilateral mode, moreover, assistance is given by the healthy limb which actively performs the tasks aiding the impaired one. The active-assistive modality provides assistance through the robot only when the patient cannot complete the exercise. In the active modality, the subject has gained the ability to independently perform tasks and the robot is used only as a means for the accomplishment of the therapy or as a monitoring device. The resistive mode challenges the ability of the patient to achieve tasks, by providing forces that oppose to the movement, either through the robot or through the healthy limb.

**Table 1:** Description of the modalities of training

Modality	Description
*Passive*	*(U/B)* The robot guides the movement and the limb passively follows
*Assistive*	*(U)* Continuous assistance is provided through assistive forces towards a target or *(U/B)* gravity support. *(B)* The unimpaired limb assists the paretic one. In both cases, active intentions are detected
*Active-assistive*	*(U/B)* The patient actively starts the movements and the robot intervenes providing assistance only when the subject is not able anymore
*Active*	*(U/B)* The patient actively accomplishes the task and the robot works as monitoring device
*Resistive*	*(U)* The robot/*(B)* the unimpaired limb provides forces that challenge the movement performed by the patient

The considered studies have been classified according to the previous modalities, either exploiting the category explicitly reported in the respective paper or assigning the study to one of the categories based on the description reported in the intervention protocol. The modalities, expressed in the papers with a different label with respect to the ones used in Table 1, have been associated with the respective category of the presented study based on the way how the task is carried out. The current analysis investigates the use of the training modalities together with the latency of the patient to detect possible relationships able to improve the results achieved through the therapy. Previous studies are present in literature that link the two sides of the coin [1, 6], but the current work aims at giving its own interpretation, as well as at extending these studies involving more applications in the bilateral mode and updating the state of the art. The previous works, although relevant, focused only on a type of robotic structure, they did not give much space to bilateral applications or they considered few studies for the different poststroke stages. The possibility to include further experiments to the analysis with respect to past investigations is surely eased by the huge advances in technology. This is also one of the reasons why the usage trends of specific approaches may have evolved over time.

With the need of updating the state of the art by including the previous mentioned characteristics, this literature study begins with an insight into the methodologies involved in the research. The analysis paragraph describes, then, the different ways in which modalities of training are applied, in three sections related to chronic, subacute and acute stages, respectively, with specific subsections related to multiple modalities and/or phases of application. The relationships emerging between modes and stages will be highlighted in the discussion section, reporting information on the relevance of the modalities both in terms of occurrence and clinical outcomes deriving from experimental trials, with a further insight into the involved robotic devices and the significance of applications in terms of ADLs.

## Methods

**Fig. 1:**
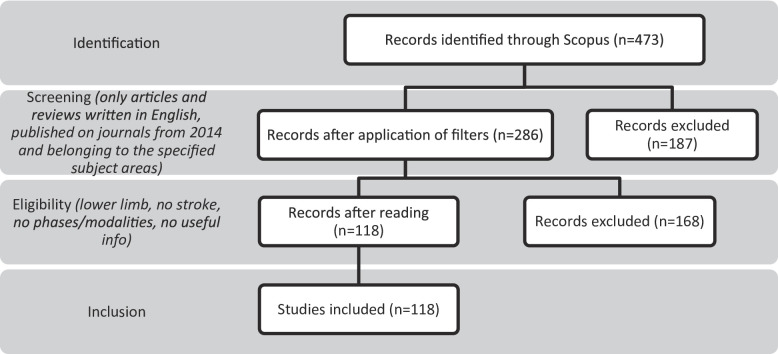
PRISMA methodology for the stages-modalities systematic search

In order to gather information pertinent to this study, a systematic literature search has been accomplished on Scopus, searching in title, abstract or keywords for papers on the relationship between poststroke stages and training modalities. The Preferred Reporting Items for Systematic reviews and Meta-Analyses (PRISMA) methodology was exploited, using the following string (also provided in the Additional file 1) « robot* AND rehabilitation AND “upper limb” AND stroke AND (training OR therapy) AND (acute OR subacute OR chronic) », which resulted in 473 documents. The search was then refined restricting the results to texts from 2014 onwards, related to the fields of medicine, neuroscience, health professions, engineering and computer science. Only articles and reviews published on journals and written in English were retained. This filtering process led to 286 papers in the screening phase. The eligibility phase was then dedicated to a general reading of the documents. Titles, abstracts and full texts were analysed to check for their relevance with respect to the research question. 168 papers were excluded because of their irrelevance with respect to the aim of the proposed work for one or more of the following reasons: the topic was related to lower limb rehabilitation, neuromotor issues different from stroke were considered, no relationship with phases of recovery or modalities of training was found or it was unclear, or a comprehensive reporting of clinical trials was not present. Finally, 118 documents have been included in the presented survey. The PRISMA methodology exploited for the analysis of interest is summed up in Fig. 1:

## Analysis on applications of the training modalities in the poststroke stages

**Fig. 2:**
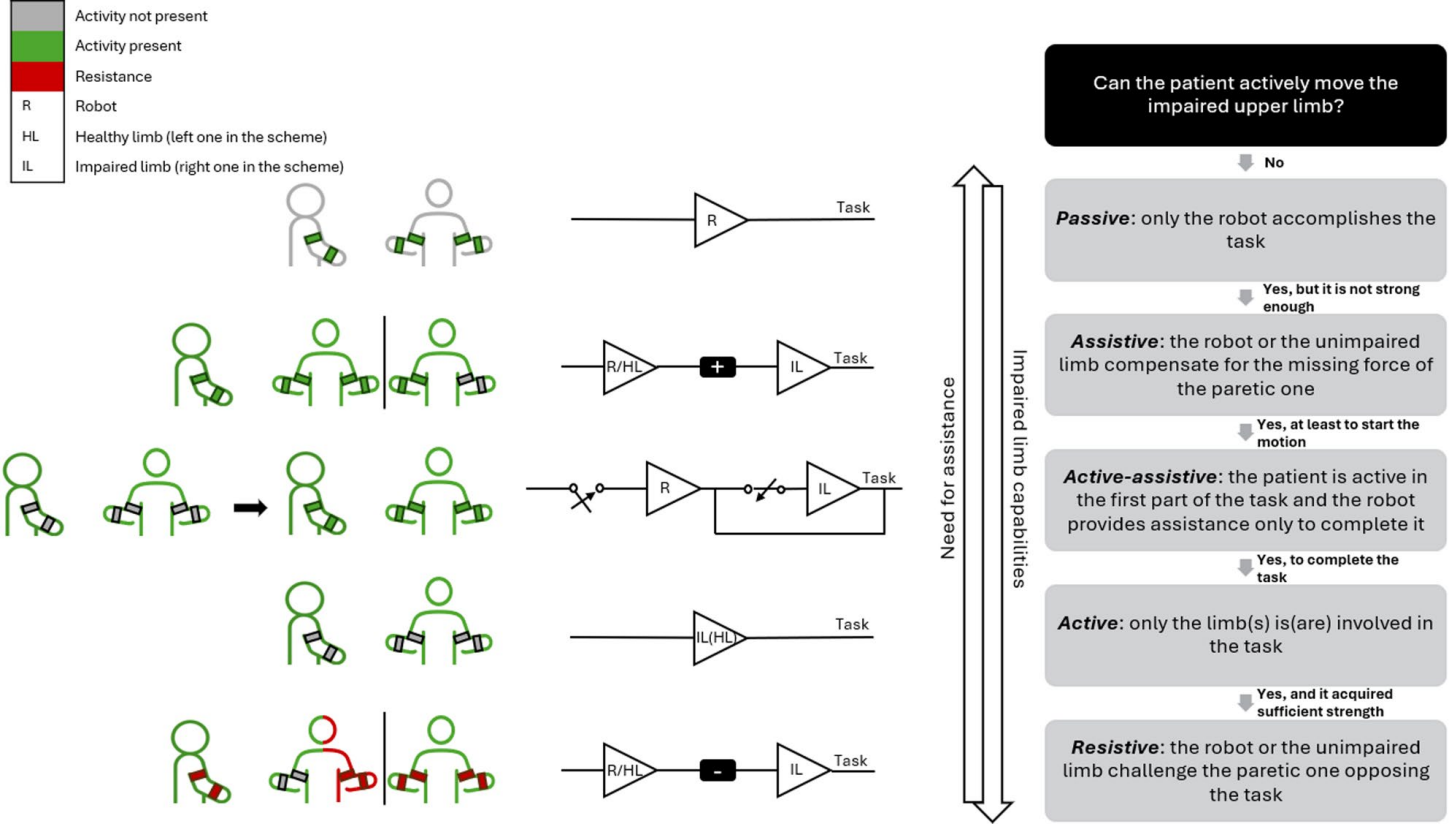
Training modalities considered in the study for both unilateral and bilateral applications

Before proceeding with the analysis of the considered works for the systematic review, a summary diagram of how the training modalities have to be interpreted in the present work is reported in Fig. 2 for unilateral and bilateral applications:

The analysis is accomplished considering the poststroke phases with decreasing number of occurrences, hence starting from the chronic stage.

### Chronic phase

Starting from the passive modality, widely diffused is the use of Continuous Passive Motion (CPM), applied in [16–18]. It is based on the repetition of passive activities that, in the case of [17] regard reach, grasp-release and flexion-extension movements and in [18] exercises related to the Degrees of Freedom (DoFs) of the shoulder, hence flexion-extension, abduction-adduction, internal-external rotation. This approach leads to improvements in Fugl-Meyer Assessment (FMA) in all the considered cases, in Motricity Index (MI) in [16, 18] and in Functional Independence Measure (FIM) in the first one. CPM contributes to provide an “automatic” relearning, hence not spontaneous/thoughtful, meaning that the patient may benefit from that procedure without an active participation. Another way of applying the passive modality during the chronic phase is for analysis purposes, as described in [19]. In this study, the patient experiences disturbances towards which he/she is not requested to react, with the only aim of monitoring the brain activity through the electroencephalography (EEG) signal. A 6-DoF exoskeleton is applied in [20] for passive movements of the 3 DoFs of the shoulder, elbow and wrist flexion and forearm supination. This application, in association with botulinum toxin A injection (BTX-A), leads to improvements in the motor control of the upper limb. Progresses are obtained in FMA, Wolf Motor Function Test (WMFT) and Motor Activity Log (MAL) in [21].

Decreasing the level of dependence of the patient from the robot, the assistive modality is met. In this paper, the term “assistance” is intended to describe a modality in which continuous support is provided to the patient but, at the same time, voluntary effort is detected. This is the distinctive characteristic between “assistive” and “passive” assistance. In the involved studies, the modality is considered assistive if the patient receives gravity support, for counteracting the weight of the impaired limb or if the system provides assistive forces towards a specified target, to help the subject in reaching exercises, as an instance. A similar approach is followed for the bilateral training, adding to the category exercises involving active and passive participation of unimpaired and impaired limb, respectively. This training is in turn considered assistive, since the active involvement of the healthy limb contributes to an increasing brain plasticity, thus stimulating the muscles of the impaired limb, as a sort of mirroring strategy [22].

Before starting with the overview on the applications of the assistive modality, some words have to be spent, in relationship with the term “as-needed”. “Assistance-as-needed” means that the robot aids the patients only as much as they need, without providing more help than necessary, being excessive support not beneficial [9]. This term is typically used in the field of rehabilitation robotics, but it is often associated to different interpretations. Firstly, it is used to highlight the ability of a rehabilitative device to switch among various modalities of training, based on the state of the patient; consequently, “as-needed” actually involves the use of multiple modalities, from passive to active and it is not related just to assistance alone [23]. The term is used also in a similar way to “active-assistive”, indicating that assistance is given only if the patient, who independently starts the task, is not however able to complete it and needs support [24]. In other cases, “as-needed” is related to pure assistance, just at different levels. Some examples with this last meaning are shown below since, for the aim of the systematic analysis, the term is used only with this last sense, to avoid misunderstandings in the definition of the training modalities. In all the other cases, the study is associated with the respective implicit mode.

The studies involved in this paper include the different kinds of assistance previously described. Counterbalancing is provided in [25], for helping the patient in reaching and grasping tasks. The support is delivered as-needed, guaranteeing continuous assistance, but with different weights based on the state of the subject. Clinical and functional improvements are detected through FMA and Bimanual Activity Test (BAT). A similar approach is exploited in [26], where assistance is as-needed in the sense that, during the reaching part of the exercises, the distance is adjusted based on the progress/degradation of the patient’s capabilities. Therefore, “as-needed” in the present work can be explained, in other words, as assistive applications in which the patient-robot initial conditions for the accomplishment of a task may change, but not the way how assistance is provided during the exercise. This approach improves motor learning but, since progresses are not transferred to ADLs, the authors suggest the association with other restorative interventions, such as brain stimulation techniques, to increase performances not occurring in a natural way. Gravity compensation is applied in [27] showing its usefulness also in triggered-control approaches. These techniques usually use a reference signal to monitor the patient’s effort during a task and they are generally associated with an active-assistive modality, because of their working principle. In this case, however, since the patient, continuously receives antigravity support by the robot, when needed, the study has been defined as assistive, because of the further help received, that may reduce the required effort, hence resulting in a different reference signal, with respect to the one that would be obtained during an unsupported training. Rehabilitation based on ADLs, such as grasping, reaching and objects transportation is accomplished in [28], comparing conventional therapy and robotic therapy, obtaining improvements on FMA and Box and Block Test (BBT), especially in the robotic group. The peculiarity of this application consists of the fact that, even when the exercises are focused on the distal extremity, which is the most difficult to recover, a positive impact of the therapy on the recovery of the proximal arm is obtained as well. The better efficacy of robotic therapy with respect to the conventional one is supported also in [29], through gravity-supported exercises focused on shoulder abduction, elbow supination and wrist extension. The application, also exploiting Virtual Reality (VR), brings positive outcomes on spasticity and motor functions, as reported by WMFT, Action Research Arm Test (ARAT), Range of Motion (RoM) and Motor Assessment Scale (MAS). Gravity compensation is applied also for bilateral tasks [30], providing support to the affected limb and improving FMA, WMFT and MAL.

Assistance provides both gravity support and assistive forces aiming at task completion in [31]. With the robot providing support against gravity, the patient must reach a target and assistive and corrective forces are generated in order to make the subject follow the correct trajectory. A similar approach is exploited in [32], where the robot provides minimum continuous assistance in a modality called *follow-assist*. Assistive forces are provided in [33] for reaching exercises. VR and neuromuscular electrical stimulation (NMES) are also employed, obtaining improvements in FMA, but not on Stroke Impact Scale (SIS) and BBT. Assistance is provided in form of torque in [34], where the robot designed for hand rehabilitation generates torque for helping the patient in the extension of fingers, reducing disability (FMA) and improving performances (ARAT) also proximally. The results, however, are not maintained over time. In [35], both proximal and distal rehabilitation is accomplished, providing assistance with NMES and gravity support. Improvements in terms of RoM related to ADLs are obtained. Moreover, distal rehabilitation improves also the proximal arm, confirming the previous considerations. Assistance is provided in [36] in a bilateral fashion, through two assistive modalities defined as *asynchronous* and *synchronous*. In both cases, the healthy limb generates the movements to be followed by the impaired one with the help of the exoskeleton, with the difference that, in the first case, the tasks are preprogrammed, whereas in the second one, the exercises have to be accomplished simultaneously for both limbs.

The following level involves the active-assistive training modality. The main method through which this modality is implemented is with the involvement of triggered-control strategies. These methodologies are based on the monitoring of a reference signal that, after the reaching of a prespecified threshold, makes the robot switch behaviour. So, the patient starts to independently execute the task and, when he/she is not able to complete it, corresponding to the reaching of the threshold, a trigger is generated and the robot starts to provide assistance. After the trigger, in most applications, assistance is generated through NMES [37–40], leading to voluntary motor recovery, better muscular coordination and reduction of spasticity (FMA, ARAT and MAS), as well as improvements in WMFT [41, 42] and FIM [42]. Even in this case, in correspondence of a distal application, progresses on the proximal limb are obtained, also due to the compensatory behaviours of chronic patients. In [43], instead of NMES, Functional Electrical Stimulation (FES) provides assistance after the trigger. Besides that, a different approach exploits EEG signals for the detection of motor imagery, exploited to monitor the patient’s intentions and to provide assistance in [44] and, similarly, in [45] and [46]. This approach leads to improvements in FMA and ARAT and it helps in maintaining the progresses related to ADLs. In the case study of [47], mechanical assistance is provided after the trigger, generated when the metacarpophalangeal (MCP) joint moments of the instructed fingers are above a threshold and those of the non-instructed fingers are below it. Improvements are obtained, again, in FMA, ARAT and WMFT, also confirming the improvements on the proximal arm, even considering the distal application. Another way of exploiting this modality of training is providing assistive forces when no movement is detected, as reported in [48, 49]. In the last work, the modality is implemented in association with error augmentation (EA), a technique supporting the enhancement of neuroplasticity and motor recovery, leading to positive changes in FMA. The active-assistive modality also refers to either training tasks that have to be actively initiated by the patient and are then continued by the robot or to other tasks based on the alternation of active movements by robot and patient. This occurs again in [32], where the active-assistive modality has two levels called, in the related study, *initiated* and *step-initiated* modes. In the first one, the task requires voluntary movement, at the beginning; after that, assistance is provided by the robot. In the second one, voluntary movements and robot-dependent movements are alternated to complete the training. Similarly, the study conducted in [50] provides both the classical triggered approach with continuous assistance by the robot after the trigger, and another one with continuous interaction between robot and patient, in order to always require effort by the subject. In the studies [51, 52], on the contrary, robotic active-assistive rehabilitation is applied together with BTX-A injection leading to improvements in FMA, ARAT and MAL, as well as a reduction of spasticity. Positive results in terms of spasticity are obtained also through robot therapy associated with transcutaneous auricular Vagus Nerve Stimulation (VNS), useful for the activation of cortical networks and applied in [53]. From a bilateral point of view, the active-assistive modality is used in [36] providing gravity support only when the patient cannot accomplish the task anymore and replicating the movements carried out by the unaffected arm.

Without the robot providing any assistance, the active modality is obtained. Most of the related studies are based on the execution of different activities that have to be accomplished freely by the patient. In [54, 55], the tasks regard mainly reaching movements, with the addition of drawing tasks in the first one and flexion-extension training in the second one, leading to improvements in motor functions in [54]. The active modality is applied also in [32], where it is defined as *free mode*.

The resistive modality is implemented with the robot challenging the patient. The opposition towards the movement is obtained through resistive spring-like forces in [56, 57] and in form of disturbances to the patient in [19] where, contrarily to the application of the same procedure during the passive mode, in this case, the subject has to face these disturbances to maintain a certain wrist torque. The study conducted in [58] can provide resistance by lifting, hence inclining upwards the table supporting the patient’s arm. From a bilateral point of view, resistance is provided by the robot to both arms in [59] and by the unaffected arm in [60], which the study calls *master arm*, opposing to the impaired one that is referred to as *slave*. In the last work, various resistive approaches are presented. Among them, one training task involves the master arm pushing the slave that, in turn, has to resist maintaining the same position. In another task, both arms have to exert the same force in order to maintain a sort of equilibrium.

#### Considerations on applications with multiple modalities

As already emerged in some considered studies, there are approaches involving multiple modalities. The bilateral study in [36] presents assistive and active-assistive modalities leading to an overall improvement of FMA, MI, FIM and Barthel Index (BI). The study in [56] can provide assistive or resistive forces, bringing positive outcomes in FMA and reduction of spasticity in proximal muscles. Besides challenging the patient, the application in [58] is also able to assist the subject inclining downwards the table; these assistive and resistive approaches result in improvements of the RoM. These two modalities are applied bilaterally in [60], improving FMA and WMFT. In [55], besides the active modality already described, passive and active-assistive modes are possible and the approach leads to improvements in muscular strength and activation. The experiments in [32] are based also on passive and active modalities, in addition to assistive and active-assistive, but without leading to relevant results in terms of FMA and ARAT. Passive and active-assistive modes are applied in [50], improving FMA and MAL and reducing fatigue during ADLs execution.

### Subacute phase

Fewer studies have been conducted relating to the subacute phase. However, also this stage presents various experimental trials for all training modalities.

The passive mode is exploited in [24], where it is denoted as *robot-in-charge* mode and in [61], in which the tasks are mainly based on grip and passive movements. Furthermore, the passive mode is applied in [62] following the same protocol used during the chronic phase in [32], with the modality defined as *guided*. Reaching exercises are reported in [63], whereas patients of study [64] were requested to complete goal-directed exercises, planar reaching tasks involving elbow and shoulder, and movements from a central target towards various lateral ones. In study [65], the passive mode is applied bilaterally with the Reha-Slide and the Bi-Manu-Track devices.

As far as the assistive modality is concerned, most of its applications are based on the use of a counterbalancing support [7, 66–69]. The exercises are based on: (i) reaching tasks [7, 69], leading to improvements in FMA, MI and MAS; (ii) virtual games and ADLs [66], improving FMA; (iii) 3D movements of the arm, i.e. flexion-extension, pronation-supination and adduction-abduction [67] and moving objects and lateral elevation [69], with progresses on motor functions. An arm support is provided also in [70] in association with games engaging elbow and shoulder, for a higher involvement of the subject. Task difficulty can change, allowing games in 1D, 2D or 3D, resulting in an approach with positive outcomes on FMA, Stroke Upper Limb Capacity Scale (SULCS) and reaching distance. Assistance is also provided through spring-like assistive forces, as reported in [57] and together with BTX-A, also during this phase, in [71]. Other two studies [36] and [62], use the same protocol followed during the chronic phase, described previously again in [36] exploiting *asynchronous* and *synchronous* modes and then in [32], through *initiated* and *step-initiated* modes, for a bilateral and unilateral rehabilitation, respectively. Unilateral and bilateral therapy is provided also by the Diego device exploited in [72], supporting the arm(s) in 3D movements of the shoulder. Bilateral assistance is reported, again, in [65], through the Bi-Manu-Track and Reha-Slide robots.

Active-assistive applications also exploit some techniques of interest in the chronic stage too, which are the ones in [36] and [62], similarly to what is reported in [32] for the chronic application. Furthermore, also in the subacute phase, triggered-based strategies are used for this kind of training, such as in [73]. In [74], after the generation of the trigger, NMES and mechanical assistance in form of constant speed are provided, improving FMA, ARAT, MAS and FIM, regaining functionalities on the whole arm. A similar method is faced in [75], but, in this case, the trigger activates FES. The experiment consists of a bilateral approach, in which the impaired hand wears a leather glove with metal tendons and the unimpaired one wears a second glove with sensors for copying the movements to the other hand. FES is activated once the fingers reach an intermediate degree of extension. Relevant changes in SIS are detected. Similarly to the chronic phase, also in this stage a variant of application for triggered methodologies is related to Brain-Computer Interfaces (BCI) and to the use of EEG to detect motor imagery, as reported in [76]. The analysis of the recorded signals shows correlation between cortex activation and motor recovery of the upper limb. In [24], the active-assistive modality is defined as *patient-in-charge* mode and it provides assistance to the subject only when he/she cannot be independent anymore. Similarly to the chronic application in [52], BTX-A is used in an active-assistive modality also during the considered phase, leading to improvements in FMA, especially in patients with mild injury [77], whereas FES is exploited together with the Rheo Therapy System in [65]. A different approach described in [78] considers a glove for fingers flexion-extension, exercises of finger-thumb opposition and rotation of the metacarpophalangeal joint. By controlling the current flowing in the coil, the glove generates magnetic forces assisting the patient, improving the results on FMA, WMFT and ADLs. Active assistance is provided in [79], both unilaterally and bilaterally, through two kinds of therapy, defined as *patient-centered* and *robot-centered*. The first one is focused on ADLs, whereas the second one aims at the accomplishment of movements of the arm’s joint. The first one has better effects on ADLs, showing the relevance of involving the patient. Considering again the study in [72], the other robots employed are able to provide also the active-assistive modality. Motore and Amadeo are unilaterally used for elbow and shoulder, and for flexion-extension of fingers, respectively, whereas Pablo can be used both unilaterally and bilaterally for 3D movements of shoulder, elbow and wrist.

Regarding the active modality, the same approaches exploited during the chronic phase are applied also during the subacute stage in [54] and [62]. Besides that, the study [80] proposes active rehabilitation of upper limb and trunk, obtaining improvements in FMA and WMFT, whereas it can be applied bilaterally, as reported in [65], with Bi-Manu-Track and Reha-Slide.

The same considerations apply for the resistive training, in which the movements against resistance accomplished in [61] have been experimented also during a later stage of latency. The resistive modality can also be applied bilaterally in this phase, as stated in [65], through the MOTOmed Viva2 device.

#### Considerations on applications with multiple modalities

The device in [71] can also provide passive, active-assistive and active modalities, besides the assistive one already cited, leading to overall improvements in the hand strength and ADLs, reducing spasticity and improving neuroplasticity. The different devices exploited in [72] lead to progresses in the ability of accomplishing movements, even though there are no particular advantages with respect to conventional therapy. Among the four involved robots, unilateral passive and active-assistive modes and unilateral and bilateral assistive and active modalities are involved. In addition to the passive modality, the experiment in [63] also provides active assistance, improving FMA especially in patients with moderately severe impairment. Besides passive mode, also assistive, active-assistive, active and resistive modalities can be provided in [64], obtaining progresses in FMA, MAS and RoM, maintained over time. The analysis in [65], providing passive, assistive and active bilateral modalities through the Bi-Manu-Track, leads to improvements in the arm impairment, after exercises focused on pronation-supination of the forearm and flexion-extension of the wrist. Remaining on bilateral approaches, the Reha-Slide implements bilateral passive, assistive and active modalities for elbow and wrist flexion-extension and shoulder abduction-adduction, with positive outcomes on BBT. The Rheo Therapy System combined with FES provides active-assistive and active modalities improving FMA.

#### Chronic vs. subacute stages

Concerning the study in [36], the improvements already described in the chronic stage remain valid also during the subacute one. The same applies for the study [54]. The assistive application in [28] is applied in both stages with improvements in FMA and BBT, both proximally and distally, even though the exercises are distal. In [57], being able to provide assistance and resistance both in subacute and chronic phases, improvements in FMA, ARAT and FIM have been reported, whereas the experiments conducted in [67], show no relevant progresses with respect to conventional therapy. Greater differences between the two stages are detected in the following studies. The same experiments conducted in both phases with passive, assistive, active-assistive and active modalities lead to irrelevant clinical progresses in the chronic phase, besides some motor abilities, suggesting the task-specific nature of these improvements [32]. Such outcomes lead to the necessity of an as-needed assistance during the subacute one [62], providing only the right level of support to the subject, in order to reduce spasticity. Furthermore, the study indicates the active-assistive modality as the most suitable one for the activation of the sensorimotor cortex. In the application of [73], electrical stimulation (ES) presents better efficacy in the subacute phase, with respect to the chronic one. All the modalities, from the passive up to the resistive one, are reported in [81], whereas passive and active modalities in [82]. In [81], considering that chronic patients are usually less impaired than subacute ones, the effort on the end-effector results indeed greater; that is why it is suggested for rehabilitation to focus more on compensatory mechanisms for the chronic patients and motor relearning approaches for the subacute ones, instead. On the other hand, cortical reorganization is stronger in subacute patients [82].

### Acute phase

This stage is the one with the smallest number of applications, being this the stage with the highest degree of impairment.

The passive modality, as an instance, is applied in [65] with the NeReBot robot. The assistive modality, on the contrary, is applied in [68] and [83] providing gravity support for the upper limb, in the second case also sustaining the trunk; assistance is provided bilaterally in [84]. Some applications of the active-assistive modality are employed too, such as in [65], again with the NeReBot. In addition, flexion-extension exercises of the elbow and ADL-based tasks are accomplished in [85], leading to improvements in MAL, FIM and BI.

#### Considerations on applications with multiple modalities

As already emerged above, some of the considered studies involve multiple modalities. The NeReBot, cited in [65] provides passive and active-assistive modalities for shoulder abduction-adduction and elbow flexion-extension and pronation-supination, improving FMA. In [84], besides bilateral assistance, unilateral passive and active-assistive modalities can be provided obtaining improvements in manual dexterity (BBT), ability of accomplishing functional tasks (WMFT), social participation of the patient (SIS) and ADLs.

#### Chronic vs. acute and subacute vs. acute stages

No particular distinctions can be done among these three phases in terms of clinical results, since the presented outcomes are referred to the entire therapy, which comprises multiple recovery phases. The study in [86] applies assistive and resistive modalities in both acute and chronic phases reporting general improvements in FMA. The same consideration applies for the assistive modality applied for acute and subacute stages in [68].

## Relevance of the training modalities based on the poststroke stage

In this paragraph, all the studies included in the presented survey, comprising those presented in the previous section, are analysed in terms of frequency of interest in the scientific community and clinical relevance deriving from trials. The frequency of usage or occurrence, in the proposed work, is used to quantify the level of interest for a specific approach in the scientific research community. The frequency of usage could represent an indicator of the efficacy of an approach, and this is demonstrated by the change in trends over time, meaning that specific procedures are preferred with respect to others because of their relevance. Nonetheless, since frequency reflects research interest/feasibility and may not map to clinical uptake or effectiveness, this occurrence study is supplemented by an analysis of clinical trial outcomes. This approach serves to validate whether high interest scores correlate with actual therapeutic success.

### Analysis of the occurrence of training modalities

**Fig. 3:**
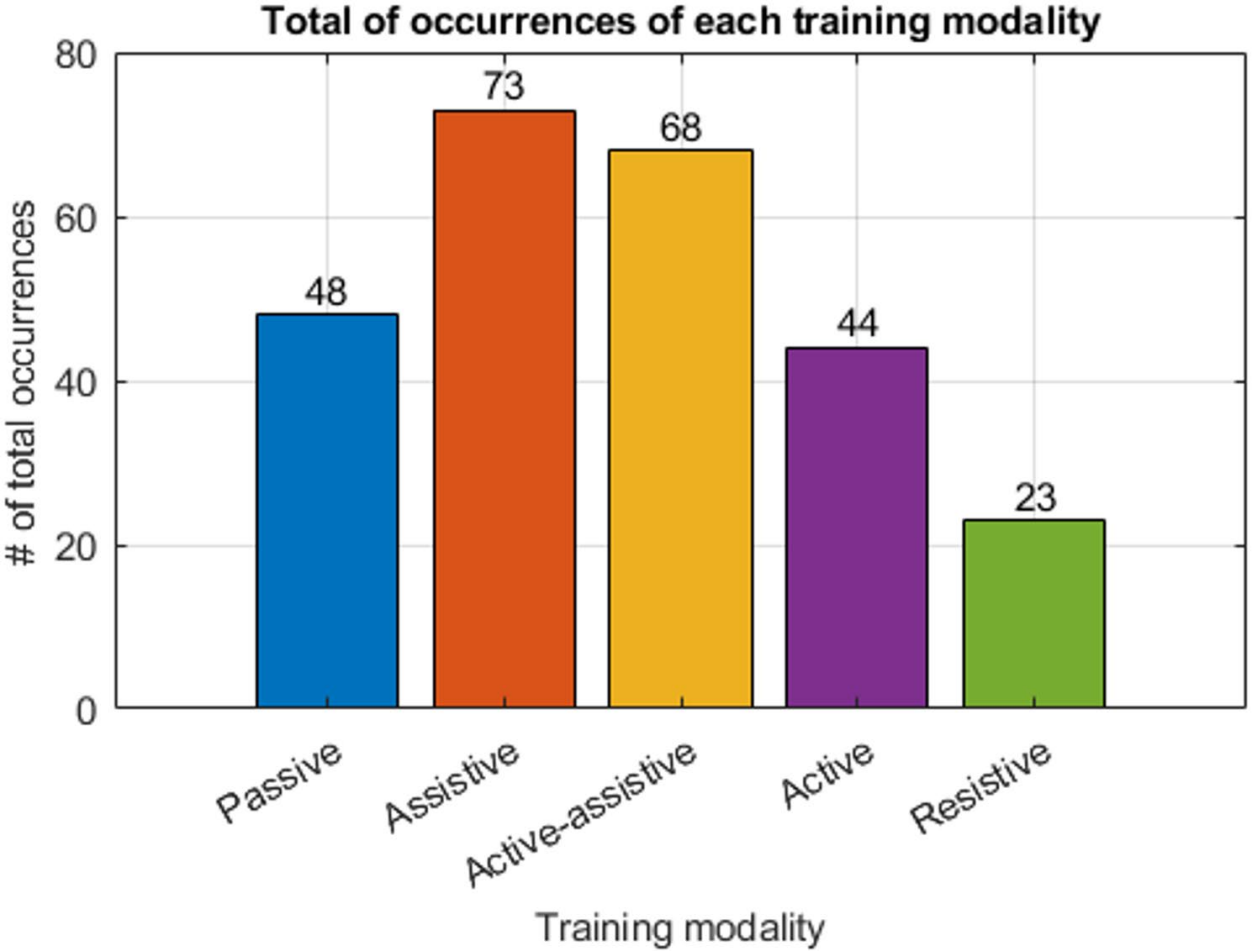
Total of occurrences of each training modality

**Fig. 4:**
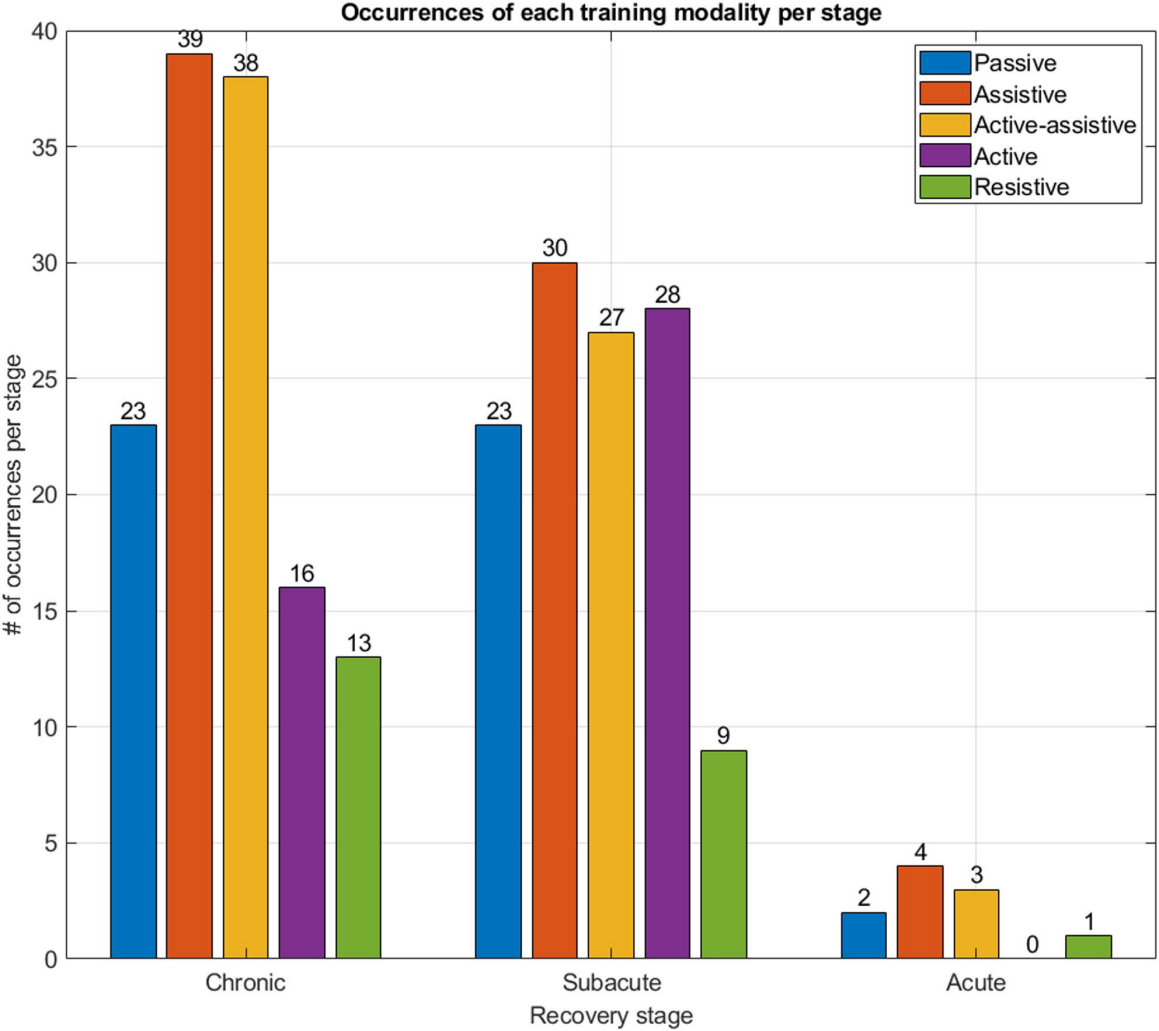
Occurrences of each training modality per stage

In order to investigate the occurrences of the different training modalities in each of the recovery phases, a total of 118 documents has been analysed for the proposed survey, including works [87–132] in addition to the ones already reported in the previous sections (further information on the studies is reported in the Additional file 2). Considering that some of the reported studies involve the use of multiple modalities, the results shown in Fig. 3 and in Fig. 4 are obtained:

It is evident that, in general, the assistive modality is the most used, followed by the active-assistive and the passive ones. Going into details of the related poststroke stages, this trend is maintained for chronic and acute stages whereas, besides the assistive modality, the other two positions change for the subacute one, seeing active and active-assistive modalities in second and third place, respectively. The resistive mode appears as the least used in the chronic and subacute phases, while the active one is never used for acute patients in the considered studies. The fact that the cooperative modalities are the most frequent ones highlights the importance of the continuous active involvement of the patient, changing the level of assistance provided and always interacting with the robot. The presence of the active modality in the top three modes in terms of occurrence for the subacute phase could be related to the ease of relearning due to the high brain plasticity of this phase, whereas the passive modality in the chronic stage could be used for an indirect, mechanic relearning, being neuroplasticity reduced in this later stage.

**Fig. 5:**
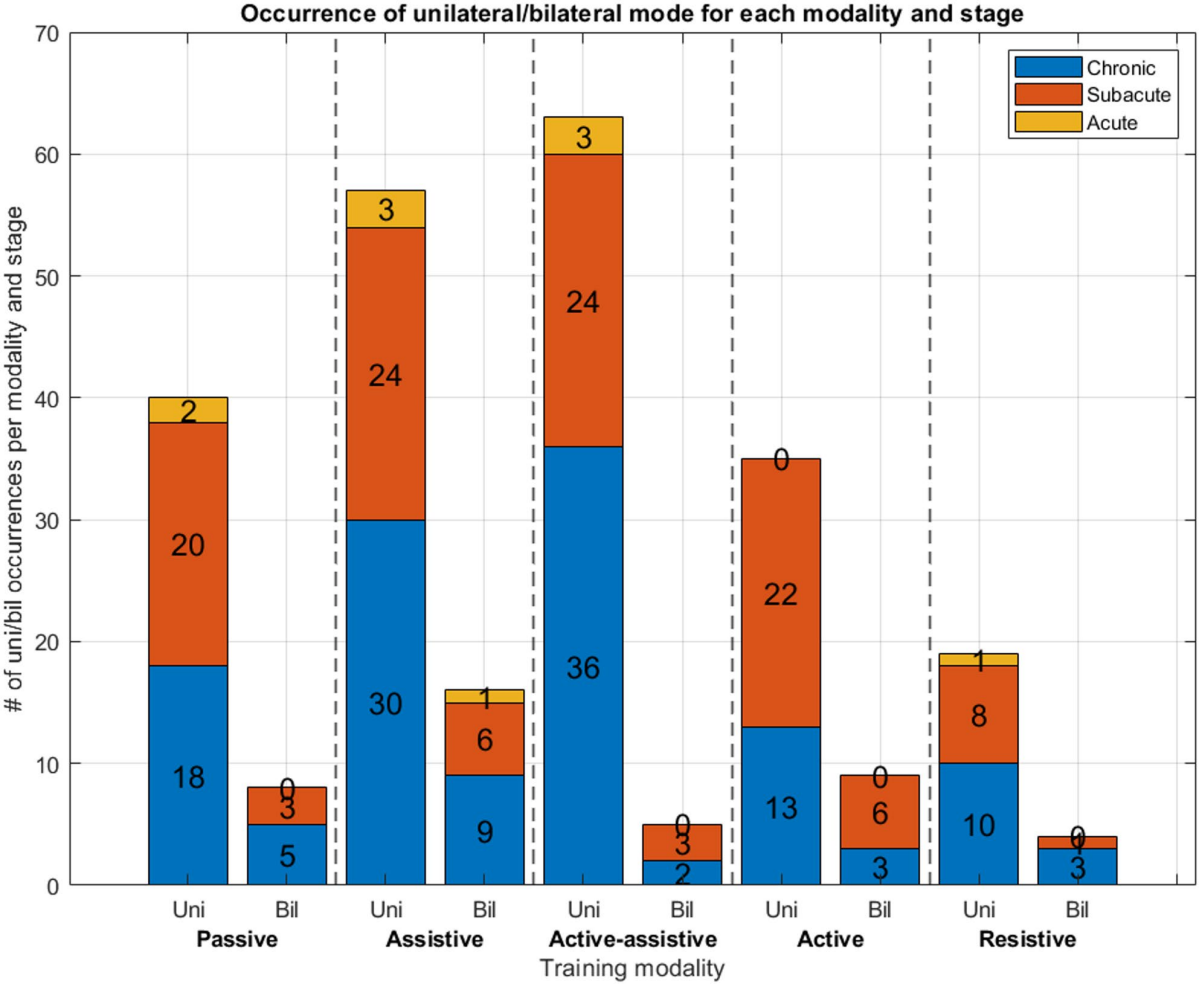
Occurrence of unilateral/bilateral mode for each modality and stage

In addition, an analysis is done also on the rates of unilateral and bilateral training. The results are summarized in Fig. 5.

Clearly the unilateral mode is more frequent. The active-assistive, assistive and passive modes prevail unilaterally, whereas the assistive, active and passive ones bilaterally. Moreover, while there are occurrences of the bilateral mode for each modality of training in subacute and chronic phases, the acute stage is the only one experiencing bilateral approaches only in one mode, that is the assistive one. This could be explained by the high impairment related to this early stage. However, the fact that the assistive mode is preferred to the passive modality for a bilateral training in this stage could be linked to the will of exploiting, also in this case, neuroplasticity and natural recovery characterising this phase. The frequency of application is equal for the passive modality in subacute and chronic patients. The first ones see mostly unilateral experiments, while the second ones exploit more a bilateral training. This could be due to the fact that, since bilateral training increases excitability of motor cortex [133], this might be a further help during the chronic phase to compensate for the reduced plasticity. Regarding the active-assistive modality, instead, even though the chronic phase sees a majority of trials with this mode, the subacute stage prevails bilaterally in terms of applications.

### Analysis of the effective relevance of the training modalities

In terms of effective relevance of the modalities of training for the poststroke stages, starting from the chronic phase, the assistive modality emerges as the one with the most significant clinical results obtained from trials. During this stage, brain plasticity is strongly reduced; therefore, ES together with triggered strategies proves efficient for brain reactivation and overall clinical scores. Outcomes are enhanced if this way of intervening is associated with antigravity support, that is why the term “assistive” was used. The association of ES and counterbalancing contributes to obtaining progresses in terms of RoM of ADLs [35]. The same consideration is done in [26], where gravity compensation alone does not improve ADLs and a suggestion is done on introducing the use of brain stimulation approaches.

For what concerns the subacute phase, the active-assistive approach proves the most useful one. The previous consideration is supported in [62], where the active-assistive mode is said to be able to activate sensorimotor cortex, during this stage. Moreover, triggered strategies based on ES lead to clinical improvements also related to ADLs [74] and the study conducted in [73] shows the better efficacy of ES in subacute patients with respect to the chronic ones. The same considerations are supported also in studies [134, 135] and, in the last one, besides NMES, Transcranial Direct Current Stimulation (TDCS) is suggested as a useful approach during this phase, with progresses as big as twice those of the chronic patients, indicating the relevance of natural recovery in this early stage. Assistive approaches, applied as-needed, also prove effective during this stage, both in the sense of progressive multiple modalities and in the case of various levels of assistance. Going back to study [62], the application of modalities with increasing difficulty proves, in fact, useful during the subacute stage, but not in the chronic one described in [32].

Consequently, mechanical approaches flanked by ES are the most successful during the chronic phase, whereas bio-based training and exercises actively involving the patient are more effective during the subacute one.

Regarding the acute stage, the number of studies involved is too restricted to be able to draw some relevant conclusions. Nevertheless, the assistive modality emerges as the most widely employed one, always leading to positive outcomes in the considered analyses.

#### Analysis on the robotic devices employed in the experiments

To clearly understand how each structure employed could have influenced the rehabilitation process, a general recap on exoskeletons and end-effectors is developed.

**Fig. 6:**
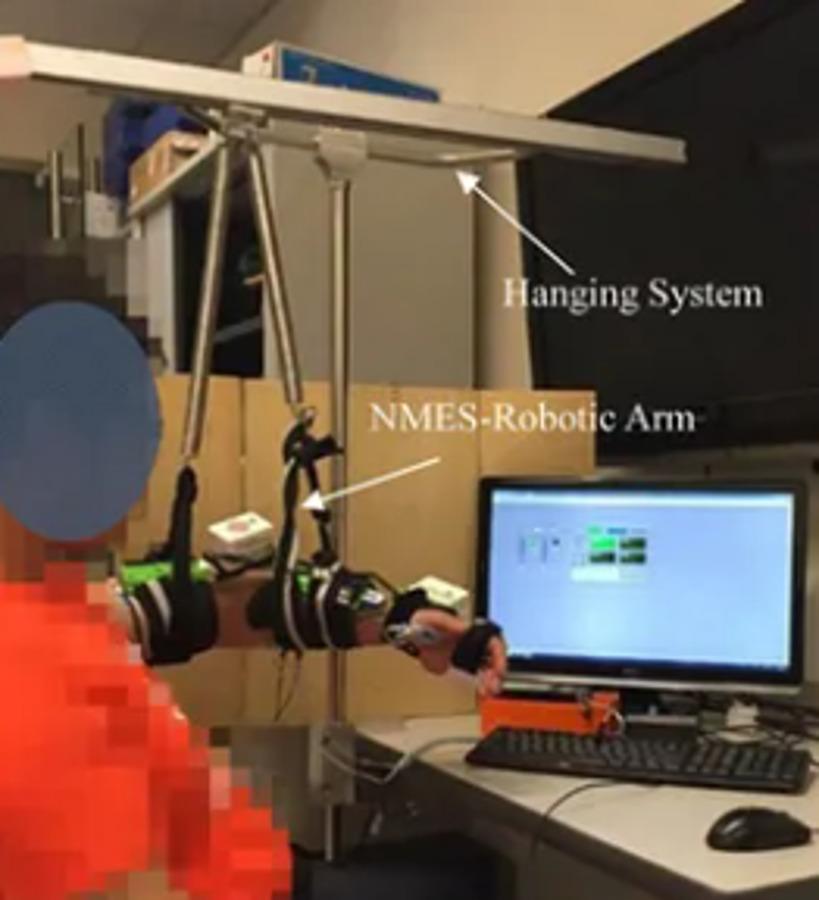
Structure of an exoskeleton [74]

**Fig. 7:**
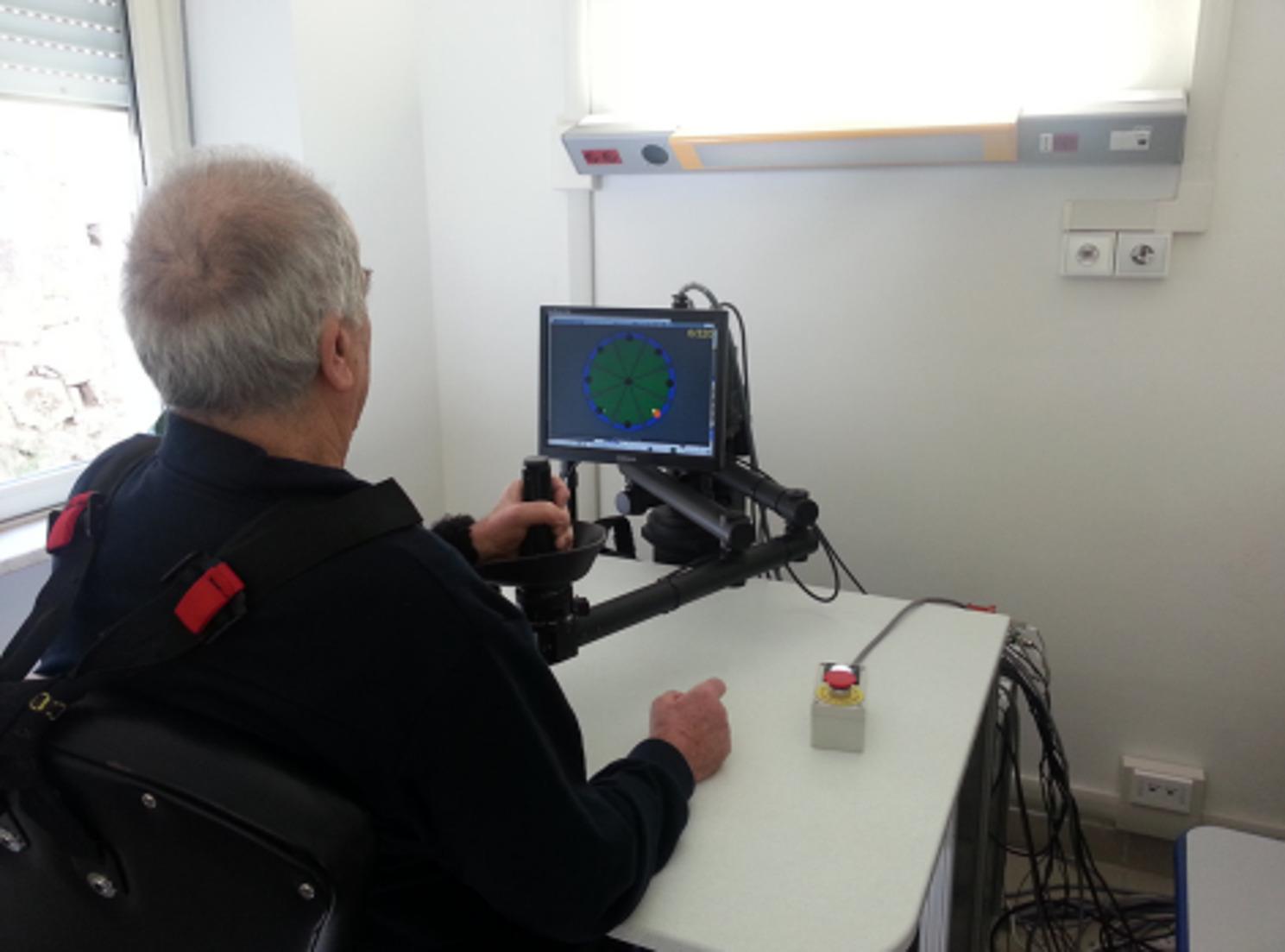
Structure of an end-effector [7]

**Fig. 8:**
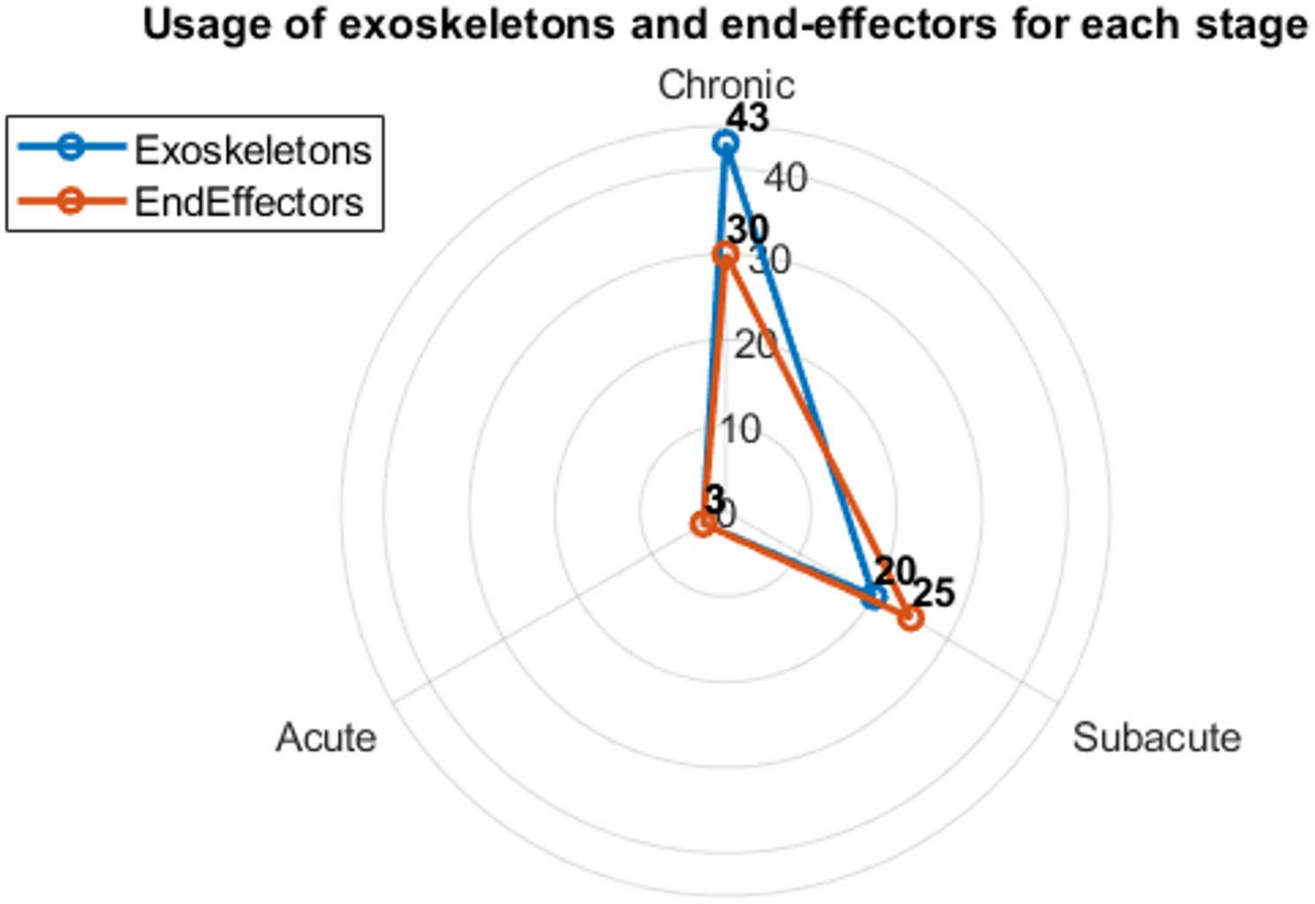
Usage of exoskeletons and end-effectors for each stage

**Fig. 9:**
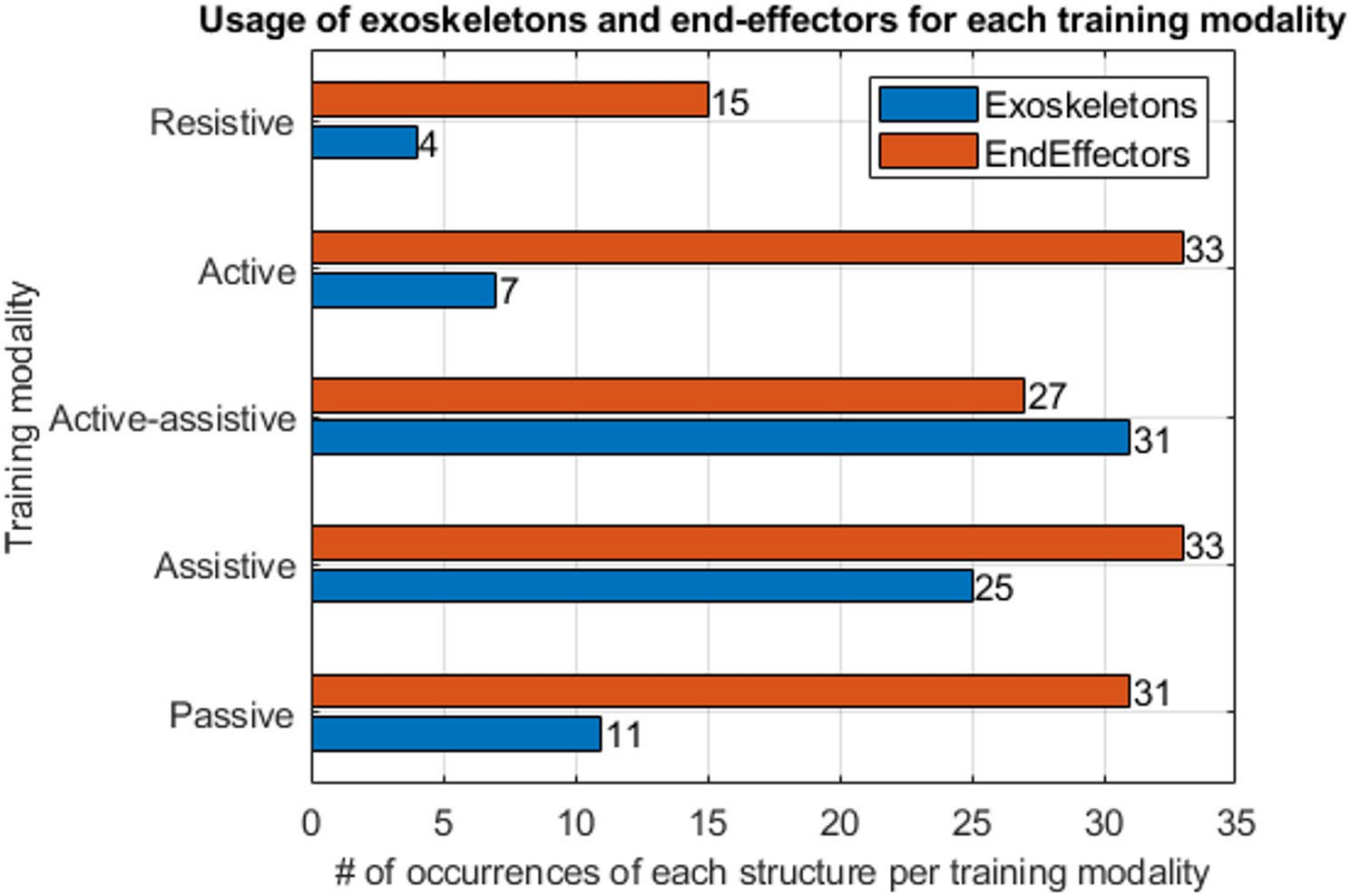
Usage of exoskeletons and end-effectors for each training modality

Exoskeletons are bionic structures encapsulating the patients’ upper limb. This gives the possibility of monitoring single joints and focusing on specific muscles, ensuring a complete control of the movement [136], but making the device hardly adaptable and hence expensive [15]. An example of exoskeleton is reported in Fig. 6:

On the other hand, end-effectors are simpler to implement and hence economic, being composed of a series of mechanisms moving the upper limb from its distal part. In this way, more freedom of moving is left to the patients, being the robot not able to monitor their posture, but making it suitable for subjects with different characteristics. The structure of an end-effector is reported in Fig. 7:

An insight on the involved robotic devices can now be given as well (Figs. 8 and 9), after categorising them in the two above-mentioned classes: exoskeletons and end-effectors. The same considerations in terms of analysis of frequency and of outcomes are maintained in this section.

#### Analysis of the occurrence of the robotic structures

The usage of the structures is reported for each of the studies considered in the proposed work:

The chronic phase sees a majority of exoskeletons applied for rehabilitation, while end-effectors are slightly predominant during the subacute stage. No significant difference in terms of usage is detected in the acute phase. An overview of the usage of the structures can be given also as a function of the training modalities. Considering that various experiments involved multiple modalities, the results are reported in Fig. 9:

It is immediately apparent that end-effectors are predominant in the majority of training modalities, confirming their suitability for several scenarios: in all cases, except for the active-assistive one, end-effectors prevail. Moreover, it emerges that for active and resistive modalities, in which more freedom of motion is left to make the patients completely rely on their neuromotor capabilities, exoskeletons are hardly chosen. While for intermediate levels of assistance, exoskeletons and end-effectors are closer in usage, noteworthy is also the high occurrence of applications of end-effectors for the passive modality.

#### Analysis of the relevance of the robotic structures

In general, in terms of clinical relevance, exoskeletons prove more beneficial for the recovery of ADLs and functionalities [85] and most of the approaches with positive outcomes on ADLs in the proposed studies use exoskeletons. However, some of the considered early-stage approaches exploited end-effectors improving activities. Consequently, if used during pre-chronic stages, end-effectors could be beneficial too and that is probably the reason why they are the most used during the subacute stage.

#### Influence on ADLs

In terms of ADLs, bilateral approaches and exercises able to recover both proximal and distal arm result effective in all stages, showing that the capability of accomplishing activities can be regained also during a later stage. Distal approaches are particularly effective for the recovery of ADLs, improving not only hand/wrist, but also shoulder/elbow, most of times making progresses even before the distal part. Since the accomplishment of activities of daily living requires a sufficient functioning of the entire upper limb and, in some cases, its collaboration with the other arm, bilateral and distally-proximally effective exercises are more suitable, also training simultaneously both extremities.

#### Relevant outcomes emerging from studies

To sum up, even though the assistive modality is prevalent in all poststroke recovery stages, patients involved in experimental trials appear to benefit more from assistive approaches with ES and gravity compensation during the chronic phase, in which the possibility to rely on high neuroplasticity has vanished. Active-assistive approaches employing ES or, in general, bio-signal triggered strategies lead to major improvements during the subacute stage, if one looks at the medical scales used for the evaluation, suggesting higher benefits deriving from the higher neuroplasticity. Regarding the acute stage, it is difficult to separate the effects of a single modality having the studies presented multiple modalities together, with a report on overall results.

In general, further relevant information that can be deducted from studies is that patients may benefit from distally-focused exercises for a recovery of both distal and proximal arm, improving ADLs. Daily activities result particularly improved also in correspondence of bilateral tasks and when exoskeletons are employed, conclusion that can be extended to end-effectors, if applied during earlier stages.

## Limitations

The reviewed studies suggest that outcomes reported in clinical scales may differ across modality–stage combinations. There are, however, some relevant factors that could potentially have further influenced the clinical outcomes and which are not directly included in the analysis, such as duration and frequency of the therapy, as well as the level of impairment of the patients, which could vary from light to severe and would likely require more intensity and slightly different approaches [137]. Moreover, in some studies involving multiple training modalities, it was not possible to distinguish the individual effect of each approach, since overall metrics were reported. In particular, no relevant conclusions have been drawn concerning the acute stage, because of the reduced number of studies. Lastly, this analysis comes from studies and trials reported in literature; it would be interesting to enrich the emerged conclusions with information deriving from daily clinical practice.

## Conclusions

The proposed work aims at linking together two variables influencing poststroke recovery, i.e. stages of recovery and training modalities. From the analysis, it emerged that trends of interest changed over time and novel methodologies have been developed enriching the field of robotic rehabilitation. For this reason, the need of an update of the categories defining the training modalities emerged and a proposal has been presented in this work for a clear classification of the ways how human-robot interaction can be achieved, incorporating both unilateral and bilateral applications. The analysis of interest in the scientific community has been flanked by an investigation of the actual relevance of the employed approaches, documented by clinical scales evaluating the experimental outcomes. From these metrics, it was deducted that some correlations are present between therapy modes and poststroke stages, sustaining that outcomes may be enhanced when the association of training modality and poststroke recovery stage is optimized. The associations which resulted to be the most effective, together with further significant information emerging from experimental trials, have been emphasized for an increased focus, to provide clinicians with a dataset of information on how the rehabilitative robotics field is evolving, providing insights on scientific findings. Conclusions presented in this work may need to be supported by application in daily clinical practice.

The proposed survey did not allow to always link obtained clinical results to exploited training modes, since, in some cases, applications exploiting multiple modalities were reported along with a general description of the overall outcomes, without assessing the influence of each individual mode. Moreover, only few studies are related to the acute phase; consequently, deeper analyses need to be done and included to extend these results. In addition, further factors are present that may have influenced the outcomes, which have not been directly considered, such as intensity of the exercises and severity of impairment. Future developments of the proposed work may involve an extension of the study to the other relevant elements.

For future works, the paper suggests the definition of more homogeneous studies, in order to obtain comparable results, either fixing the poststroke stage and experimenting different modalities or vice versa, exploiting the same approaches.

## Supplementary Information

Supplementary Material 1. Keywords for the search query.

Supplementary Material 2. Analysis of the studies included in the review.

## Data Availability

All data generated or analysed during this study are included in this published article and in the following supplementary information files: Additional file 1 (.pdf) and Additional file 2 (.xlsx).
