# Supplementary material for: On the correlation between training modalities and recovery stages in poststroke robotic rehabilitation of the upper limb: a systematic review
Source: J Neuroeng Rehabil. 2026 Apr 30;23:197. doi: 10.1186/s12984-026-02005-7 (PMC13285516; doi:10.1186/s12984-026-02005-7)
Supplement: Supplementary file 1 — Supplementary Material 1. Keywords for the search query. [file 12984_2026_2005_MOESM1_ESM.pdf]

Disorder

1. stroke

Intervention

2. robot*
3. rehabilitation
4. #2 AND #3

Body segment

5. “upper limb”

Combination disorder, intervention and body segment

6. #4 AND #5 AND #1

Limitations

7. training
8. therapy
9. #7 OR #8
10. acute
11. subacute
12. chronic
13. #10 OR #11 OR #12

Total search strategy

14. #6 AND #9 AND #13 → robot* AND rehabilitation AND “upper limb” AND stroke AND (training OR therapy) AND (acute OR subacute OR chronic)
